# Actigraphic registration of motor activity reveals a more structured behavioural pattern in schizophrenia than in major depression

**DOI:** 10.1186/1756-0500-3-149

**Published:** 2010-05-27

**Authors:** Jan O Berle, Erik R Hauge, Ketil J Oedegaard, Fred Holsten, Ole B Fasmer

**Affiliations:** 1 Haukeland University Hospital, Department of Psychiatry, P.O.Box 23 Sandviken, N-5812 Bergen, Norway; 2 Department of Clinical Medicine, Section for Psychiatry, Faculty of Medicine and Dentistry, University of Bergen, Norway; 3 Olaviken Psychiatric Hospital, Bergen, Norway

## Abstract

**Background:**

Disturbances in motor activity pattern are seen in both schizophrenia and depression. However, this activity has rarely been studied objectively. The purpose of the present study has been to study the complexity of motor activity patterns in these patients by using actigraphy.

**Findings:**

Motor activity was recorded using wrist-worn actigraphs for periods of 2 weeks in patients with schizophrenia and major depression and compare them to healthy controls. Average motor activity was recorded and three non-parametric variables, interdaily stability (IS), intradaily variability (IV), and relative amplitude (RA) were calculated on the basis of these data. The motor activity was significantly lower both in patients with schizophrenia (153 ± 61, mean ± SD, p < 0.001) and depression (187 ± 84, p < 0.001), compared to controls (286 ± 80). The schizophrenic patients had higher IS and lower IV than the controls reflecting a more structured behavioural pattern. This pattern was particularly obvious in schizophrenic patients treated with clozapine and was not found in depressed patients.

**Conclusions:**

Motor activity was significantly reduced in both schizophrenic and depressed patients. However, schizophrenic patients differed from both depressed patients and controls, demonstrating motor activity patterns marked by less complexity and more structured behaviour. These findings may indicate that disturbances in motor activity reflect different pathophysiological mechanisms in schizophrenia compared to major depression.

## Findings

Assessing motor activity is essential in every psychiatric evaluation. Despite this, objective methods are rarely used in psychiatric clinical practice.

It is well known that increased or decreased gross motor activity is often seen in patients with schizophrenia [1]. Altered motor activity is also an integral part of the clinical picture of depressive states [2], and is seen in seasonal affective disorder [3] and major depression in children and adolescents [4]. Depressed patients differ from normal and psychiatric comparison groups with regard to objectively quantified gross motor activity, body movements, speech, and motor reaction time [5]. Psychomotor retardation during depression may be measured by actigraph [2]. In major depressive disorder hypofrontality and negative symptoms are related [6]. Total motor activity of patients with schizophrenia recorded by actigraphy has been found positively correlated with structural brain correlates (volume of left anterior cingulate cortex) [7], and quantity of movement has also been related to negative symptoms. However, no correlation between the motor symptoms of the Positive and Negative Syndrome Scale (PANSS) and the actigraphy results has been reported [8].

Circadian rhythm desynchronization may play a key role in mood disorders, and a therapeutic goal in treating depression and other mood disorders is to restore normal circadian rhythms. Actigraphy is more reliable than sleep logs in the study of sleep and circadian rhythms [9]. In schizophrenia possible circadian rest-activity rhythm disturbances have not yet been widely studied.

In a study of schizophrenia by Wirz-Justice and colleagues [10] patients treated with clozapine had remarkably highly ordered rest/activity cycles, whereas the patients on classical neuroleptics had minor to major circadian rhythm abnormalities. This was the first documentation of circadian rest-activity cycle disturbances in schizophrenia related to class of drug.

The diagnosis and classification of Schizophrenia and Mood disorders are based on the fulfilment of symptomatic criteria, defined by the International Classification of Disease (ICD) and DSM-IV respectively, and in both cases diagnoses consists of a combination of traits and/or symptoms defining a very broad syndrome. In both disorders diagnoses are characteristically based on the patient's description of symptoms and objective, quantitative findings would be particularly helpful in the diagnosis of these disorders.

Our aim was to explore the usefulness of objective recording of motor activity to reveal disturbances in the pattern of motor activity in schizophrenia and major depressive disorder, as this could reflect differences in the underlying pathophysiological mechanisms of these disorders.

## Methods

### Subjects

The study group consisted of 23 open ward long-term patients with schizophrenia, and 23 currently depressed patients, five of those inpatients (28%). Characteristics of the patients with schizophrenia are shown in Table 1, and with major depression in Table 2.

**Table 1:** Demographic and clinical characteristics of patients with schizophrenia (n = 23).

Age (mean ± SD)	46.7 ± 10.9 (range 27 - 69)	
Year at first hospitalization	24.4 ± 9.3 (range 10 - 52)	
		
	N	%
Gender (male)	20	87
Diagnostic subtype		
Paranoid	18	78
Non paranoid	5	22
BPRS	50.6 ± 8.9 (n = 18)	
clozapine	55.0 ± 4.9 (n = 8)	
not clozapine	47.0 ± 9.9 (n = 10)	
p = 0.04 (t-test)		

**Table 2:** Demographic and clinical characteristics of patients with a major depressive episode (n = 23).

Age (mean ± SD)	42.8 ± 11.0	
Education (years ± SD)	11.5 ± 2.8	
MADRS (start of registration, mean ± SD )	22.7 ± 4.8	
MADRS (end of registration, mean ± SD )	20.0 ± 4.7	
	N	%
Gender (male)	13	57
Married or cohabiting	11	48
Currently working	3	13
Diagnoses		
Unipolar major depressive disorder	15	65
Bipolar I disorder	1	4
Bipolar II disorder	7	30

Control subjects were hospital employees (n = 23), students (n = 5), and patients without serious medical or psychiatric symptoms from a primary care office (n = 4). The control group comprised 20 women and 12 men, average age 38.2 ± 13.0 years (mean ± SD), range 21 - 66. None of the control subjects had a history of mood or psychotic symptoms.

All the patients in the schizophrenia group were on antipsychotics, adherence to medication confirmed by therapeutic drug monitoring. Of these patients, eight men and one woman were treated with clozapine, the others with traditional and atypical antipsychotics (Table 3). The clozapine treated patients were slightly younger (43.3 vs. 48.9 years, p < .05), and had higher BPRS scores (Table 1). The rationale for using clozapine was to improve symptom control, both positive and negative symptoms. Some of the clozapine patients had been refractory to several alternative regimens. There was no tendency of patients suffering from more pronounced negative symptoms being treated with clozapine. All 23 patients have a chronic severe mental illness and were considered unable to live independently.

**Table 3:** Psychotropic drug treatment for patients with schizophrenia (n = 23).

	N	%
Clozapine	9	39
Other antipsychotics	14	61
Traditional antipsychotics	6	26
Atypical antipsychotics	8	35
Mood stabilizers	5	22
Clozapine dose	481 ± 218 mg (range 300 - 900)	
Serum level of clozapine	1671 ± 1164 μmol/l (range 326 - 3333)	

Fifteen of the 23 patients with major depression were on antidepressants, some co-medicated with lithium, mood stabilizers, antipsychotics, anxiolytics or hypnotics, while eight patients did not use psychotrophics.

### Diagnostic Procedures

Diagnostic assessments of mood disorders were performed by one of the authors (OBF), assessments of schizophrenia were made by another author (JØB), based on a semi-structured interview (SCID-I, section A: affective episodes, section B: psychotic and associated symptoms) and DSM-IV criteria (American Psychiatric Association 1994) [11].

### Evaluation of depressive and psychotic symptoms

In the depression group affective symptoms were assessed by Montgomery-Asberg Depression Rating Scale (MADRS) scores [12], and in the schizophrenia group symptoms were assessed by the Brief Psychiatric Rating Scale (BPRS) [13].

### Recording of motor activity

Actigraphy is a quantitative method both to measure gross motor activity, and to examine diurnal variations in motor activity. In our study motor activity was monitored with an actigraph worn at the right wrist (Actiwatch, Cambridge Neurotechnology Ltd, England). The right wrist was chosen for the participant's convenience. Previous studies have shown small differences between the right and left wrist [14-16]. Total activity counts were recorded for one minute intervals for a continuous period of two weeks. Both patients and controls were instructed to wear their actigraphs at all times except when taking a shower.

### Mathematical analyses

The variables interdaily stability (IS), intradaily variability (IV), and relative amplitude (RA), developed for analysis of actigraph data, were used [17]. The IS quantifies the invariability between the days, that is, the strength of coupling of the rhythm to supposedly stable environmental factors. The IV indicates the fragmentation of the rhythm, that is, the frequency and extent of transitions between rest and activity. RA is calculated using data from the most active 10 h period and the least active 5 h period in the average 24 h pattern. IS, IV and RA were calculated for the whole two week period.

### Ethics

The study protocol was approved by the local ethics committee (REK III, Health -West, Norway).

### Statistics

T-tests (two-tailed) were used to calculate differences between groups, with a significance level of 0.05. SPSS version 15.0 was used for the statistical analyses.

## Results

**Figure 1:**
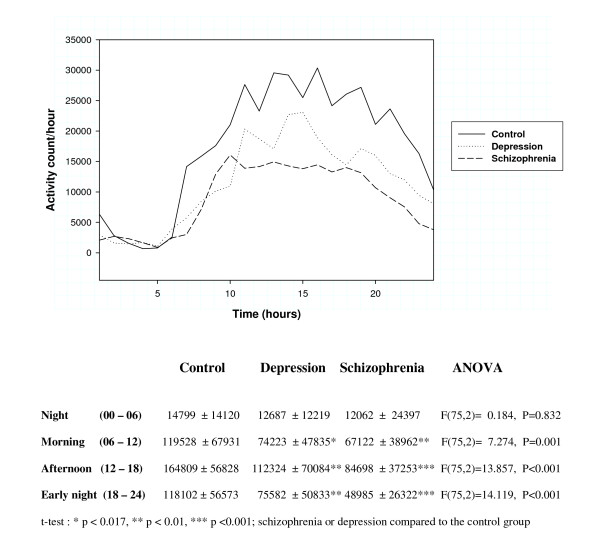
**Activity counts during 24 hours with one hour intervals (mean), and 6 hours intervals (mean ± SD)**.

Activity counts during 24 hours, calculated using one hour intervals, for patients with major depression, schizophrenia, and healthy controls are shown in Figure 1. Total and night time activity, as well as results from the calculation of interdaily stability, intradaily variability, and relative amplitude are shown in Table 4. Analyses of variance (ANOVA) showed significant differences between the groups for all these parameters, with the exception of relative amplitude. Schizophrenic patients and patients with depression both showed substantial reductions in total activity (47% and 35% compared to the control group), but the schizophrenic patients had a more pronounced reduction in night time activity (57%) than the depressed patients (31%). The value for interdaily stability was 18% higher in the schizophrenic patients compared to controls, while the depressed patients showed little difference (4% reduction). Intradaily variability was 18% lower in the schizophrenic patients and 8% lower in the depressed patients, compared to controls. In the depression group, no differences in the five parameters were seen, comparing bipolar and unipolar patients (data not shown).

**Table 4:** Actigraphic recordings in patients with schizophrenia and depression, compared to controls.

	Control	Schizophrenia	Depression	ANOVA
Total activity (00 - 24)	286 ± 80	153 ± 61***	187 ± 84 ***	F(75,2) = 22.965, P < 0.001
Activity night (23 - 06)	89 ± 53	38 ± 32 ***	61 ± 33	F(75,2) = 10.162, P < 0.001
Interdaily stability	0.446 ± 0.113	0.526 ± 0.154	0.428 ± 0.129	F(75,2)= 3.722, P = 0.029
Intradaily variability	0.901 ± 0.168	0.742 ± 0.190	0.825 ± 0.282	F(75,2)= 3.714, P = 0.029
Relative amplitude	0.837 ± 0.118	0.801 ± 0.141	0.836 ± 0.121	F(75,2)= 0.657, P = 0.521

t-test, *** p < 0.001; schizophrenia or depression compared to the control group

All results in the control groups were similar for males and females. We have therefore combined the results from male and female participants. The schizophrenic patients were mostly male (20/3), while in the control group there were more females (20/12). When restricting the ANOVA analyses to males only, differences between the groups were still significant, for average activity (42% reduction in the schizophrenic patients) and night time activity (57% reduction in the schizophrenic patients). Intradaily variability was 19% lower in the schizophrenic patients compared to controls and interdaily stability was 12% higher, but the ANOVA analyses were not significant for these parameters. Average age in the two groups was different (46.7 vs. 38.2 years, p = 0.013), but when analyzed separately in the control and schizophrenic groups, there was no significant correlations with age. The depressed patients were not significantly different form the controls in terms of gender distribution and age. We found no significant correlations between either MADRS or BPRS and the different actigraphy motor parameters.

The results from schizophrenic patients treated with clozapine, compared to those treated with other antipsychotics are shown in Table 5. There were similar reductions in activity counts in the two patient groups (46% and 47%), but the clozapine group had a more pronounced reduction in night time activity (73% vs. 47%). Only the clozapine group showed significant increased interdaily stability (38%) and reduced intradaily variability (24%). In the clozapine group the serum level of clozapine did not correlate with motor activity.

**Table 5:** Actigraphic recordings in schizophrenic patients treated with clozapine or with other antipsychotics, compared to controls.

	Control	Clozapine	Other antipsychotics	ANOVA
Activity	286 ± 80	155 ± 54 ***	152 ± 67 ***	F(51,2) = 22.127, P < 0.001
Activity night	89 ± 53	24 ± 10 ***	47 ± 38	F(51,2) = 8.887, P < 0.001
Interdaily stability	0.446 ± 0.113	0.616 ± 0.166 **	0.468 ± 0.119	F(51,2) = 7.136, P = 0.002
Intradaily variability	0.901 ± 0.168	0.685 ± 0.223 **	0.778 ± 0.163	F(51,2) = 5.898, P = 0.005
Relative amplitude	0.837 ± 0.118	0.875 ± 0.061	0.753 ± 0.158	F(51,2) = 4.317, P = 0.019

t-test, ** p < 0.01, *** p = 0.001 or <0.001; clozapine or other antipsychotics compared to the control group

As 18 of the 23 patients diagnosed with schizophrenia had the paranoid subtype, the other subgroups were too small for meaningful comparisons.

## Discussion

We found clear differences in motor activity between the three groups we have studied. However more interestingly, we found differences between patients and controls, and also between patients with schizophrenia and depression, using the two non-parametric variables interdaily stability (IS) and intradaily variability (IV), introduced by Van Someren et al [17]. The interdaily stability (IS) reflects the 24 hour rhythm component; a low value indicates reduced variability between days. The intradaily variability (IV) assesses the fragmentation of the rhythm; a low value indicates less fragmentation or more structure of the rhythm. A more structured rhythm was found in schizophrenic patients. This may reflect a more structured or monotonous behavioural pattern. Our findings of increased interdaily stability and the reduced intradaily variability in the schizophrenic patients are compatible with this. This pattern is not seen in the depressed patients even if they have a similarly reduced activity level.

To the best of our knowledge, this is the first study based on actigraphy to address differences in motor activity pattern between patients with schizophrenia and patients with depression.

The activity levels were reduced to the same extent in depressed and in schizophrenic patients, when compared to healthy controls. The marked alteration in motor activity between the schizophrenic patients and the controls [1], and reduced activity in depressed patients, is in agreement with previous studies [2,18-20]. One study reports a lower motor activity level and a reduced fragmentation of motor activity during wake in unmedicated depressed inpatients [21]. The lower motor activity reported by Volkers [21], is in agreement with our current findings. Volkers et al [22] also reports an increase in motor activity after treatment with imipramine. We do not have comparable data. Volkers et al [21] also reported increased motor activity during sleep. We were not able to replicate this. According to our study, a lower motor activity level was seen in depressed patients at night, although not significantly so. A reduced fragmentation of the rhythm during daytime as reported by Volkers [21] is difficult to compare with our results due to differences in the mathematical methods used in these two studies. Psychomotor symptoms may be more pronounced in bipolar patients [23], but we were not able to find any differences between bipolar and unipolar patients in our sample.

It has recently been demonstrated that objectively measured motor activity by actigraphy is inversely related to clinically assessed negatives symptoms in schizophrenia [8]. The low motor activity level found in patients with schizophrenia may reflect this.

Marked alterations in motor activity in depressed patients are usually associated with melancholic depression [24]. The diagnostic category melancholic depression can overlap with other diagnostic categories like unipolar depression or bipolar I disorder.

However, reduced motor activity may be a more general feature in major depression as only one of our patients had a melancholic depression.

Motor impairment in depression and in schizophrenia has been linked to the bradykinesia of Parkinson's disease, implying altered dopaminergic function, involving the nigrostriatal system [20]. The tremor of Parkinson's disease, loss of facial mobility, and walking pattern, all indicate a motor system operating in a more structured or stereotype fashion [25].

Our findings therefore suggest that the schizophrenic patients have a more severe disturbance of the motor control system than the depressed patients, perhaps more similar to that seen in Parkinson's disease.

Goldberger has suggested that diseases and syndromes can better be understood as a dynamic reordering instead of a disordering process resulting in a presentation characterized by loss of complexity and development of stereotypy, such as seen in Parkinson's disease [25,26]. We hypothesize that similar long-term actigraphic recordings in patients with Parkinson's disease may give results comparable to that seen in our schizophrenic patients.

The schizophrenic patients showed an activity pattern characterized both by low total activity and lower night-time activity, and in addition a more regular rest-activity cycle, different from both controls and from the patients with depression. In agreement with the study of Wirtz-Justice, Haug and Cajochen from 2001 [10], we found the most marked differences on motor activity in the patients treated with clozapine. These patients are from the same setting as the patients treated with other antipsychotics, expecting the same external Zeit-gebers. Zeit-gebers means environmental agents or events that provide the cue for setting or resetting a biological clock, daylight or social factors are examples of common Zeit-gebers to help us get synchronized with our environment. It is therefore reasonable to suppose that differences between the groups must be caused either by intrinsic biological differences or by the difference in drug treatment. The controls in our study were employed and working, while the patients were not. Obviously this is a potential source of bias that might contribute to increased values of motor activity in the controls. In the schizophrenia group, all were in-patients in a psychiatric institution, although some of those were living quite independent with their individual daily routines including preparing their own meals within such frames. One limitation of this study is that the interdaily stability for the majority of patients in the schizophrenia group may be under influence of the institutional schedules like meals, although the difference between clozapine treated schizophrenic patients and schizophrenic patients treated with other antipsychotics can not be explained this way.

One limitation of our study is the non-blind assessment of diagnoses. However, the methods of investigating the motor activity did not require a subjective evaluation. Our long-term patients with schizophrenia, although in a stable phase, all have a chronic severe mental illness and they may not be representative for the whole diagnostic group. At the other end of the spectrum our patients with depression are mostly out-patients and may represent a group with only low or intermediate severity of illness, as indicated by the MADRS scores. These results require replication in larger samples. We have not done a systematic assessment of adverse events, including neurological side effect of motoric type (akathisia, parkinsonism etc) in our patient groups [27]. Therefore, a limitation of our study is the possible motoric side effects in the schizophrenia group, particularly those not being treated with clozapine.

Patients and control persons wore the actigraph on the right hand. The rationale for this was that most people have their wrist watch on their left hand and we thought it would be inconvenient to have two such devices on the same arm for an extended period of 2 weeks. It is of course possible that this may have influenced the results, and inflated the difference between the controls, which were employed and working, and the patient groups. On the other hand, previous studies have shown only small differences between actigraph recordings from the right and left arms [16].

An obvious strength of this study is the long recording period, two weeks both for patients and healthy controls, making observations of rhythms richer and enhancing the detection of more nuanced and subtle changes in the groups studied. The few existing actigraph studies of patients with mental illness usually have shorter recording periods, typically 24 to 48 hours. A further strength is the recording of antipsychotic medication serum level of all patients with schizophrenia, confirming their adherence to the medication.

## Conclusions

The pattern of motor activity in schizophrenic patients is marked by less complexity, a finding different from controls and patients with depression, and this finding may have both clinical and patho-physiological importance. The difference in motor activity complexity could reflect that motor activity derangements in schizophrenia and depression have different foundations. Our work is one of the few examples describing objective registration of clinical symptoms and signs in patients with psychiatric illness. This methodology may prove useful in future studies, allowing improved symptom description and a better understanding of the complexity of these mental illnesses, in particular regarding treatment with medication.

## Competing interests

The authors declare that they have no competing interests.

## Authors' contributions

JOB, KO and OBF designed this study and provided patients and controls. ERH and FH also participated in the design of the study. The statistical analysis was provided by OBF and ERH. The coordination of the study was done by OBF and JOB. All authors participated in interpreting the results and drafting the manuscript. All authors read and approved the final manuscript.
